# Detecting functional magnetic resonance imaging activation in white matter: Interhemispheric transfer across the corpus callosum

**DOI:** 10.1186/1471-2202-9-84

**Published:** 2008-09-12

**Authors:** Erin L Mazerolle, Ryan CN D'Arcy, Steven D Beyea

**Affiliations:** 1 Institute for Biodiagnostics (Atlantic), National Research Council, Halifax, Nova Scotia, Canada; 2 Department of Psychology and Neuroscience, Dalhousie University, Halifax, Nova Scotia, Canada; 3 Department of Radiology, Dalhousie University, Halifax, Nova Scotia, Canada; 4 Department of Physics, Dalhousie University, Halifax, Nova Scotia, Canada

## Abstract

**Background:**

It is generally believed that activation in functional magnetic resonance imaging (fMRI) is restricted to gray matter. Despite this, a number of studies have reported white matter activation, particularly when the corpus callosum is targeted using interhemispheric transfer tasks. These findings suggest that fMRI signals may not be neatly confined to gray matter tissue. In the current experiment, 4 T fMRI was employed to evaluate whether it is possible to detect white matter activation. We used an interhemispheric transfer task modelled after neurological studies of callosal disconnection. It was hypothesized that white matter activation could be detected using fMRI.

**Results:**

Both group and individual data were considered. At liberal statistical thresholds (p < 0.005, uncorrected), group level activation was detected in the isthmus of the corpus callosum. This region connects the superior parietal cortices, which have been implicated previously in interhemispheric transfer. At the individual level, five of the 24 subjects (21%) had activation clusters that were located primarily within the corpus callosum. Consistent with the group results, the clusters of all five subjects were located in posterior callosal regions. The signal time courses for these clusters were comparable to those observed for task related gray matter activation.

**Conclusion:**

The findings support the idea that, despite the inherent challenges, fMRI activation can be detected in the corpus callosum at the individual level. Future work is needed to determine whether the detection of this activation can be improved by utilizing higher spatial resolution, optimizing acquisition parameters, and analyzing the data with tissue specific models of the hemodynamic response. The ability to detect white matter fMRI activation expands the scope of basic and clinical brain mapping research, and provides a new approach for understanding brain connectivity.

## Background

### White Matter fMRI Activation

The vast majority of functional magnetic resonance imaging (fMRI) studies adhere to the assumption that activation is fundamentally restricted to gray matter regions (e.g., [1,2]). Two main factors support this assertion: 1) relative to gray matter, cerebral blood flow and volume are reduced in white matter (e.g., [3,4]); and 2) the neurophysiological source of fMRI has been linked to postsynaptic potentials (e.g., [5,6]), the majority of which take place in gray matter.

However, Tettamanti and colleagues have argued that, despite the decreased perfusion relative to gray matter, there is no direct evidence against detecting white matter activation using fMRI [7]. Although it has been demonstrated that fMRI activation is more strongly linked to postsynaptic potentials than action potentials, energy dependent processes are not exclusively found in gray matter. For example, axonal conduction is often mediated by nodes of Ranvier, at which adenosine triphosphate dependent sodium-potassium ion pumps work to restore ionic gradients across the neuronal membrane after action potential propagation [8-10]. In addition, there is evidence that spiking activity is also correlated with fMRI activation [11-13], albeit perhaps to a lesser extent than the correlation with local field potentials [5]. The correlation between fMRI signal changes and spiking activity has been described as "fortuitous" (i.e., a result of the correlation between spiking activity and local field potentials). Evidence for this view is derived from work showing that selective blocking of spiking activity has a minimal effect on fMRI signal changes [14]. However, in gray matter regions where both types of potentials are generated, it is possible that larger fMRI responses associated with local field potentials overshadow the contribution of spiking activity.

In fact, a number of fMRI studies have reported white matter activation. For example, in a study of visual motion, Brandt and colleagues reported a negative signal change in the white matter of the occipital lobe contralateral to the stimulated hemisphere [15]. In another study, Mosier and Bereznaya reported corpus callosum activation as part of a distributed network involved in swallowing [16]. Of particular note, there is a body of work suggesting that white matter activation can be detected when using tasks that are designed to elicit interhemispheric transfer across the corpus callosum [7,17,18]. While the majority of these studies have used basic visuomotor interhemispheric tasks, functionally lateralized stimuli have also been used to elicit interhemispheric transfer.

### The Poffenberger Paradigm

Interhemispheric transfer was first experimentally varied using the Poffenberger paradigm [19], in which a light flash is presented briefly to a visual hemifield. Subjects are instructed to respond with either the ipsilateral (uncrossed) or contralateral (crossed) hand. Many studies have employed variants of the Poffenberger paradigm to study the dynamics of interhemispheric transfer, reporting reaction time delays of approximately 2–8 ms in the crossed condition [20,21]. Performance of split brain patients, in which the corpus callosum has been resected, was significantly delayed in crossed conditions (4–14 times longer reaction times than healthy individuals; [20,22]).

With respect to fMRI studies of the Poffenberger paradigm, Tettamanti and colleagues, Omura and colleagues, and Weber and colleagues have all reported activation in the genu of the corpus callosum (in addition to task related activation in gray matter regions) [7,17,18]. Preliminary work by our group has confirmed this finding [23], suggesting that it is possible to target the corpus callosum using a specific interhemispheric task.

### Interhemispheric Transfer and Functionally Lateralized Stimuli

One complication with the Poffenberger paradigm is that the performance of split brain patients is impaired, but not abolished, in the crossed condition. This observation suggests that alternate subcortical connections may be involved, such as the superior colliculus [24]. In contrast, other interhemispheric transfer tasks have been devised in which performance in the crossed condition is completely abolished for split brain patients (e.g., [25-31]). In general, these tasks incorporate lateralized brain processing, such as left hemisphere word processing (e.g., [32-35]) and right hemisphere face processing (e.g., [35-37]). By using visual hemifield presentation, it is possible to create crossed and uncrossed conditions.

Previously, we examined interhemispheric transfer of word and face stimuli in healthy individuals using fMRI at 1.5 T [38]. The results showed that crossed conditions were associated with an increased number of activated voxels in regions adjacent to the splenium of the corpus callosum. This study highlighted the importance of studying white matter activation but was limited in terms of the field strength and sample size.

### The Current Study

#### Experimental Design Overview

The current study expanded on the previously developed interhemispheric transfer (IT) task [38]. The objective was to examine white matter activation with 4 T fMRI using a larger sample size. Word and face stimuli were presented in the context of lexical decision and face detection tasks. Stimuli were presented to the right and left visual fields in order to manipulate visual IT. To further engage interhemispheric transfer, the task included a motor IT condition. Motor IT was manipulated by varying the response hand such that it was controlled by the hemisphere either ipsilateral or contralateral to the specialized hemisphere (words: left hemisphere; faces: right hemisphere). Thus, there were four conditions under investigation: low IT, visual IT, motor IT, and high IT (i.e., both visual and motor IT). A block design was used to maximize sensitivity to white matter activation.

#### Study Hypotheses

The key hypothesis predicted that the interhemispheric transfer task would elicit activation in the corpus callosum. In addition, differences between IT conditions (visual/motor) were explored to determine whether information about the functional organization of the corpus callosum could be delineated. Standard whole brain group level analyses were performed. Individual variability and time course data for callosal activation were also considered.

## Results

### Group Results

Behavioural validation of the interhemispheric transfer (IT) task is described in the Methods section. Unless otherwise stated, fMRI activation was evaluated using conventional statistical thresholds from gray matter research (p < 0.05, corrected for family-wise errors [FWE], extent = 2).

Activation maxima for the four conditions relative to rest (i.e., low IT, visual IT, motor IT, and high IT) are presented in detail in Additional File 1. In brief, all conditions revealed the same general pattern, with bilateral clusters spanning superior frontal, inferior frontal, medial frontal and cingulate, superior and inferior parietal, occipital-temporal, and cerebellar regions. There was no significant difference in activation maps designed to isolate interhemispheric transfer (e.g., *t*-contrasts of high IT > low IT). Based on this outcome, the conditions were combined for the remaining analyses (i.e., task > rest). The absence of a conditional effect is further considered in the Discussion section (below).

**Figure 1:**
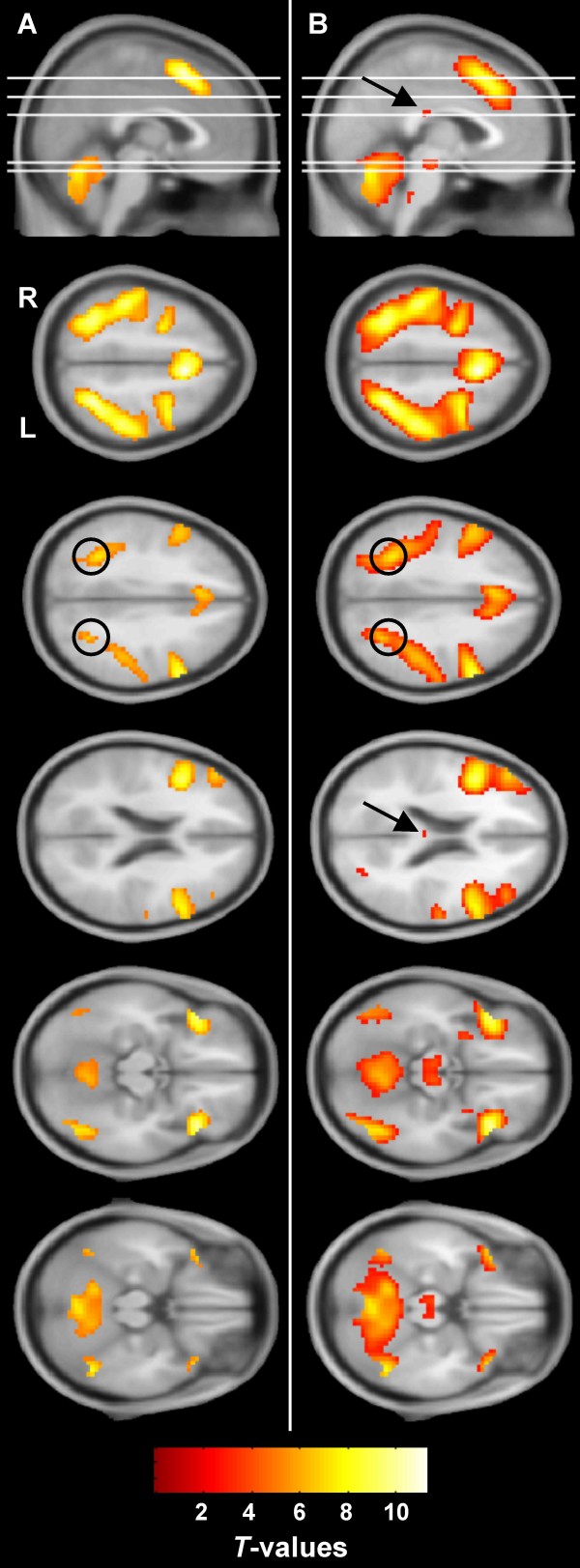
**Whole brain activation (group level)**. Task related activation for the group is presented at two thresholds: **(A) **p < 0.05, FWE corrected and **(B) **p < 0.005, uncorrected. Circles: superior parietal activation. Arrows: corpus callosum activation (detected in **B **only). White lines: axial slice positions. Scale bar represents *t*-values. R: right, L: left.

Task related activation for the group is presented in Figure 1. Not surprisingly, the same network of regions described above was identified in this analysis. While no corpus callosum activation was detected at standard thresholds (Figure 1A), activation was detected in the isthmus using liberal thresholds. At p < 0.005 (uncorrected), a four voxel cluster was detected in the corpus callosum (Figure 1B). This cluster's extent increased to 11 voxels at p < 0.01 (uncorrected). It is worth noting that the activation patterns were very similar between the different thresholds with the exception of the additional corpus callosum activation at the liberal thresholds.

### Individual Results

**Figure 2:**
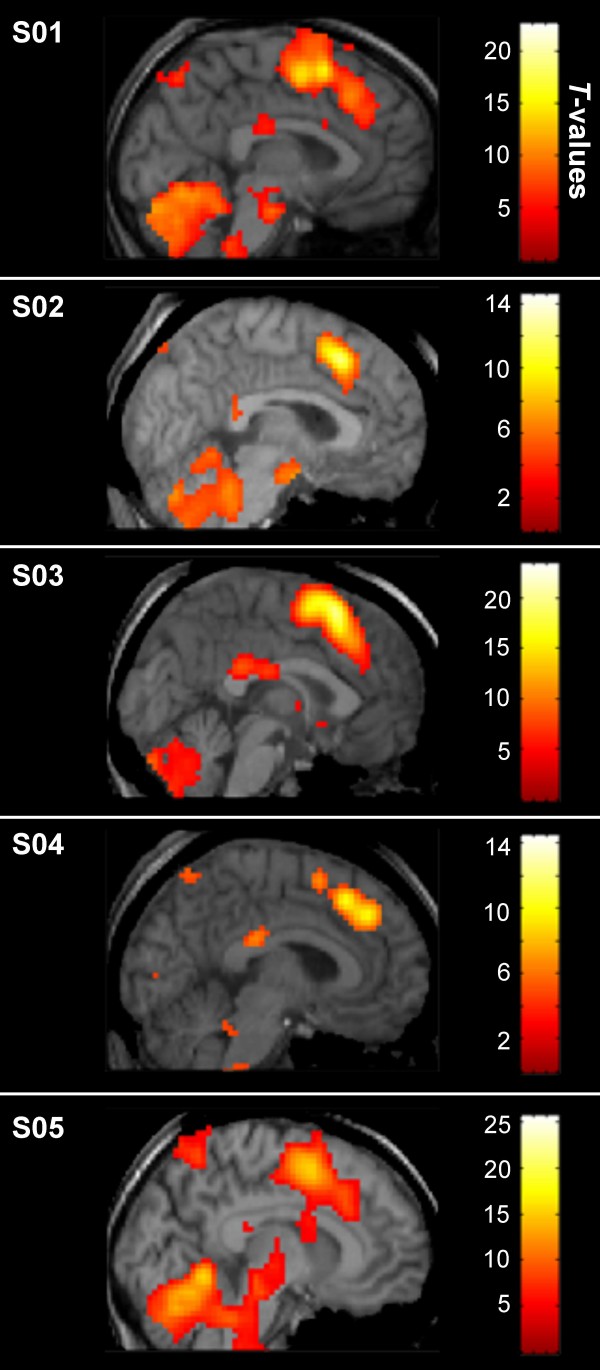
**Activation clusters located primarily on the corpus callosum (individual level)**. The five subjects with clusters located primarily within the corpus callosum are presented (whole brain activation results are shown). The isthmus of the corpus callosum was activated in four subjects, with splenium activation for S02. Scale bars represent *t*-values. R: right, L: left.

In order to detect group level corpus callosum activation, liberal statistical thresholds were necessary. However, it was possible that reduced sensitivity at the group level was due to individual variance in corpus callosum activation. To explore this further, we investigated individual level activation results. Whole brain activation was examined to identify clusters located primarily in the corpus callosum. To reduce the potential contribution of partial volume effects, clusters located primarily in gray matter were excluded, even if they partially overlapped with the callosum. This analysis revealed white matter clusters for five subjects (21%). In four subjects, these clusters were located in the isthmus, while one subject (S02) had a cluster in the splenium (Figure 2).

**Figure 3:**
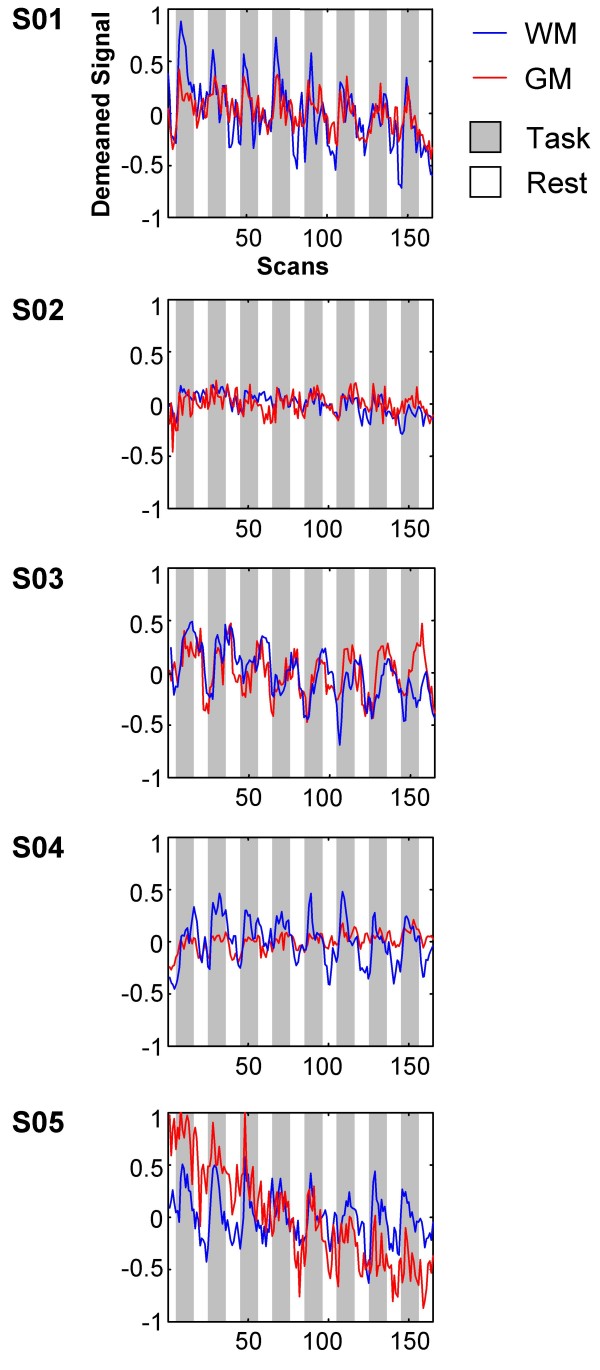
**Time course of corpus callosum activation**. Time course signals were extracted from the activated clusters that were located primarily on the corpus callosum, excluding gray matter voxels and voxels with significant movement related activity (see text and Figure 2). For comparison, time course signals were also extracted from similar gray matter clusters (Table 1). Scans (x axis) represent 2 s intervals (effective TR). Signals were extracted from all four sub-runs and demeaned to account for global signal differences. The sub-runs were averaged together for each subject. WM: white matter, GM: gray matter.

**Figure 4:**
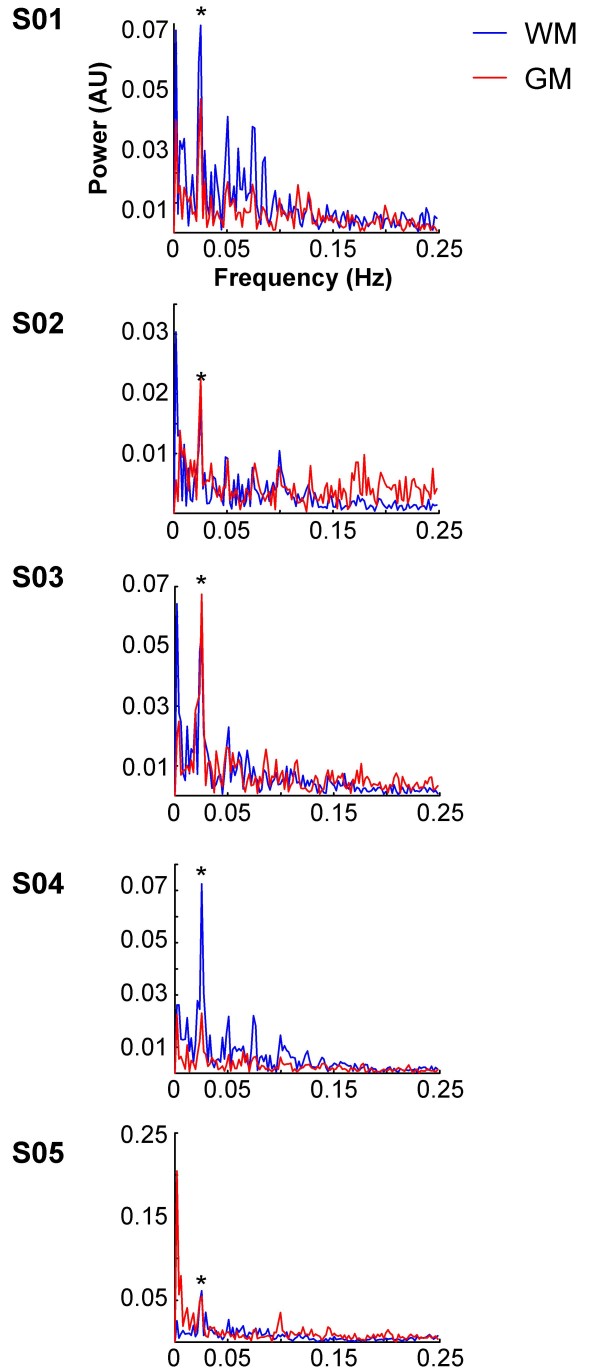
**Frequency spectra**. Frequency spectra were created from the time courses (Figure 3) using FFT. Power (in arbitrary units [AU]) is on the y axis and frequency (in Hz) is on the x axis. Note the peak in the power spectra at the task frequency (0.025 Hz) for both gray and white matter (asterisks). WM: white matter, GM: gray matter.

To verify that these clusters exhibited task related signal changes, time course data were extracted and compared to similar clusters in gray matter (Table 1). Within the callosal clusters, voxels with significant movement related signal changes were excluded (see Methods for details). Figure 3 shows the time course data for both white and gray matter relative to the task blocks. The white matter responses were comparable to those observed in gray matter. The frequency spectra for the time courses were also created (Figure 4). A peak in the power spectra at the task frequency (0.025 Hz) was observed in both white and gray matter for all five subjects.

**Table 1:** Clusters used in the time course analysis

	Corpus callosum clusters					Comparable gray matter clusters				
	MNI coordinates					MNI coordinates				
Subject	x	y	z	Cluster size	Z-score	x	y	z	Cluster size	Z-score
01	3	-21	24	41 (31)	5.54	64	-45	0	31 (29)	6.95
02	3	-39	15	12 (10)	5.44	54	-36	3	24 (20)	5.43
03	0	-27	21	116 (52)	6.06	48	36	18	927 (39)	7.04
04	0	-27	24	33 (22)	6.69	-51	-60	-18	58 (51)	5.20
05	0	-24	18	9 (9)	4.82	-39	-78	27	75 (7)	6.14

Details on the clusters from which time course signals were extracted (Figure 3). For each subject, a gray matter cluster was selected such that it was comparable in size and activation intensity to the corpus callosum cluster under investigation. Note that the cluster size column has two values: the actual size of the cluster from the whole brain analysis, and the size of the cluster used in the time course analysis (in parentheses). See Methods for details.

## Discussion

### Overview of Results

#### Validation of the Interhemispheric Transfer Task: Gray Matter Activation

In the current study, interhemispheric transfer (IT) was investigated with 4 T fMRI. At the group level, whole brain fMRI results revealed a distributed, bilateral network including parietal, frontal, cingulate, fusiform, and cerebellar regions. This network was consistent with the nature of the task, which involved word and face processing as well as motor responses. A key result was the involvement of superior parietal regions, which have been previously implicated as the cortical site of visuomotor interhemispheric transfer [11,39,40].

#### White Matter fMRI Activation

The concept of white matter activation in fMRI is controversial. Based on the current results, the existence of white matter activation warrants continued investigation. While callosal activation was not detected at the group level at standard statistical thresholds (p < 0.05, FWE corrected), it was detected in the isthmus in the uncorrected activation maps (p < 0.005). This finding is particularly promising given that the isthmus contains connections between the superior parietal cortices [41]. As noted above, the superior parietal regions have been implicated in several interhemispheric transfer studies [39,40].

Individual level analyses revealed that five subjects showed activation in the corpus callosum (21%), four with clusters in the isthmus and one with a cluster in the splenium. The time course data extracted from these clusters exhibited task related activation similar to comparable clusters in gray matter. This pattern of results suggested that contrary to current assumptions, the possibility of white matter activation could not be ruled out.

It is important to note that these findings may not generalize to all white matter structures. Indeed, the fact that activation was only detected in medial areas of the corpus callosum (rather than extending laterally towards the corresponding cortical areas) suggests that the finding may be region dependent. One explanation relates to the higher density of fibres in the medial corpus callosum (compared to lateral callosal regions): it is possible that simultaneous activity a relatively dense region of axons is required for measurable hemodynamic changes in white matter. Alternatively, there may be differences in perfusion between white matter regions. However, more work is needed to understand the regional dependence of white matter activation.

In addition, a number of issues related to sensitivity remain to be investigated. For instance, we found that the analysis parameters greatly affected sensitivity: when we did not include the movement parameters as regressors of no interest in the statistical analysis (see Methods), sensitivity to callosal activation was reduced both at the group and the individual subject level (not shown). Other issues related to sensitivity are discussed below.

### Improved Detection of White Matter Activation

#### Increasing Spatial Resolution to Reduce Partial Volume Effects

The individual level analysis was restricted to activation clusters located primarily on the corpus callosum. This reduced the potential for false positives that could arise due to partial volume effects (i.e., nearby gray matter activation contaminating corpus callosum voxels). However, the reverse is also possible. That is, small signal changes in white matter may have been lost due to contamination from nearby quiescent gray matter. In either case, smaller voxels will allow better delineation between the gray and white matter signals. As a next step, higher resolution fMRI will be applied in order to confirm activation in the corpus callosum in a greater proportion of subjects. While increased spatial resolution will reduce the signal-to-noise, the contrast-to-noise may improve due to a relative increase in signal contribution from white matter.

#### Optimizing fMRI Acquisition

In addition to higher spatial resolution, a number of other acquisition parameters could be optimized for white matter signal detection. Indeed, known differences in gray and white matter relaxation properties and hemodynamic characteristics likely affect the selection of acquisition parameters. For example, ongoing work in our lab is investigating the effect of T2 weighting, echo time, and contrast agents on sensitivity to white matter activation.

#### Modelling the Hemodynamic Response

Given the differences in cerebral blood flow and volume [3,4,42,43], it is highly likely that the white matter hemodynamic response is different from that of gray matter. We used a parametric analysis technique that modelled the hemodynamic response with canonical shape and timing characteristics. In such model based analyses, any deviations in terms of shape and timing between the predicted and observed response will decrease sensitivity (e.g., [44,45]). In this case, a data analysis technique that does not make assumptions about the hemodynamic response may be more sensitive [38]. Alternatively, an empirically derived white matter hemodynamic response function could increase sensitivity. By characterizing the hemodynamic response in white matter, it is also possible to gain insight into the underlying physiology.

### Detecting Conditional Differences in the Corpus Callosum

One potential criticism relates to the fact that the activity did not vary as a function of condition (e.g., high IT > low IT). The ability to experimentally vary the intensity and/or location of activation in the corpus callosum is a critical step for validating white matter activation. However, the use of conditional manipulations to achieve this was complicated by difficulties in designing an adequate control condition. This problem was confirmed by recent findings from the corresponding event-related potential (ERP) study. D'Arcy and colleagues used the same task and analyzed both scalp recorded ERPs and underlying current source activations. Both ERPs and source analyses revealed that interhemispheric transfer was present in both crossed and uncrossed conditions [46]. The findings suggested that it is unlikely that the cerebral hemispheres function in isolation.

The current experimental design also allowed comparisons between visual and motor IT, with the goal of activating different regions of corpus callosum. Given that there were no conditional differences in the whole brain analysis, the subsequent analyses were restricted to task versus rest (i.e., collapsed across visual and motor IT). These analyses showed activation only in posterior callosal regions (isthmus and splenium). As a result, the involvement of different regions of the corpus callosum in IT remains an open question. Studies of the Poffenberger paradigm have shown more anterior callosal activation in the genu region [7,17,18,23]. Therefore, corpus callosum activation may be task dependent. One approach may be to directly compare different tasks in order to modulate the location of corpus callosum activation.

## Conclusion

The current observation of activation in the corpus callosum provides evidence for a controversial phenomenon in fMRI research. Despite the fact that it is rarely reported, there is no direct evidence against the idea of detecting white matter activation (e.g., [7]). In the present study, there was preliminary evidence for group level white matter activation, which was subsequently detected in five subjects (21%). In addition to higher magnetic field strengths and larger sample sizes, sensitivity to white matter activation may be further increased by higher spatial resolution, optimized acquisition, and improved data analysis techniques.

## Methods

### Participants

Thirty healthy adults volunteered for an fMRI study. A self report questionnaire was used to screen participants for known psychiatric and neurological conditions and medications that could affect their performance. For all participants, English was the first language and vision was normal or corrected to normal. Informed consent was obtained prior to the experiment and all participants received compensation. Data from six participants were excluded due to technical problems with the images.

Of the remaining 24 participants (13 females and 11 males), the mean age was 24.0 years (standard deviation [SD] = 3.6, range = 19.4–33.9) and the mean level of education was 17.3 years (SD = 2.0, range = 13–22.5). The Edinburgh Handedness Inventory [47] indicated 23 participants were right handed and one was mixed handed (laterality quotient mean = 85.0, SD = 16.4, range = 28.6–100). All participants completed an exit questionnaire and were debriefed after the experiment. The study had ethics board approval.

### Experimental Design and Task

The experiment was designed to manipulate IT. Stimuli were presented to either the right visual field (RVF) or left visual field (LVF). Word and face stimuli were used to elicit lateralized processing in the left and right hemispheres, respectively. Participants were asked to respond with the right or left hand. As a result, four IT conditions were examined:

1) low IT (e.g., LVF faces and left hand response);

2) visual IT (e.g., RVF faces and left hand response);

3) motor IT (e.g., LVF faces and right hand response); and

4) high IT (e.g., RVF faces and right hand response).

Participants were instructed to maintain fixation for the duration of the experiment. Pilot eye tracking data confirmed that participants were able to maintain fixation. A central fixation was presented continuously. Stimuli were presented to the left or right of the fixation (150 ms duration), with the inside edge at least 2.3 degrees away from centre to avoid stimulating the vertical meridian [35]. For each trial, participants were asked to judge stimulus format, in which they discriminated words versus pseudowords, and faces versus scrambled faces. To keep the response requirements simple, stimulus format was indicated using the index and middle fingers (counterbalanced across subjects). Responses to stimuli of the same type (i.e., words/pseudowords versus faces/scrambled faces) were made with the same hand, depending on the IT condition (see motor IT details below). Participants were instructed to respond as quickly and accurately as possible.

The experiment was presented in a block design format, with blocks of low IT, visual IT, motor IT, and high IT conditions (eight blocks per condition). Eight stimuli (two each of words, pseudowords, faces, and scrambled faces) were presented per block. The inter-stimulus interval (ISI) was varied pseudorandomly within each block (1.85, 2.85, or 3.85 s, mean ISI = 2.6 s). Each block lasted 22 s, with an 18 s rest period between blocks.

Blocks were grouped together into two runs, with sixteen blocks per run. Due to response hand requirements, one run contained the low and visual IT blocks, and the other run contained the motor and high IT blocks. Each participant completed both runs (counterbalanced order). Response hand assignment was only changed between runs (low and visual IT run: right hand for word stimuli, left hand for face stimuli; vice versa for the motor and high IT run). Participants performed practice tasks (with feedback) at the beginning of each run to ensure adequate performance (at least 50% accuracy). Each run was divided into two sub-runs to allow participants a chance to rest (330 s per sub-run). Each sub-run started with an 18 s fixation.

Stimuli were presented using E-Prime software (Psychology Software Tools, Inc.). Stimuli were back projected onto a screen that was affixed to the front of the bore of the scanner. Subjects viewed the screen through a mirror mounted to the head coil. Behavioural data were collected using an in-house magnetic resonance-compatible button pad and recorded using E-Prime.

### Stimuli

#### Word Stimuli

Sixty-four monosyllabic, four letter words were selected from the Medical Research Council (MRC) Psycholinguistic Database [48,49]. A number of linguistic factors were controlled across conditions: imageability, concreteness, Kucera-Francis written frequency, and familiarity ratings from the MRC database; and pronunciation regularity from the Centre for Lexical Information (CELEX) English lexical database [50]. Pseudowords were derived from a second set of 64 words (matched with the real words on the linguistic factors) by replacing one or two letters. The pseudowords were manually verified to ensure they were not proper nouns or homonyms of real words. The word stimuli were presented in black uppercase letters on a white background (24-point Arial font). Words were presented vertically (e.g., [51-54]) with visual angles of approximately 3.0 degrees high by 0.9 degrees wide.

#### Face Stimuli

Sixty-four front view, expressionless, and unfamiliar faces were selected from the Max Planck Institute for Biological Cybernetics Face Database [55]. The digitized images were converted to grayscale and the image contrast was increased so that the features could be rearranged without creating the appearance of seams in the images. Scrambled faces were created by rearranging the order of the eyes, nose, and mouth within the face outline for each of the 64 faces. The eyes were never the bottom most feature to ensure that they fit within the face outline. Face stimuli occupied a visual angle of approximately 3.4 degrees high by 2.8 degrees wide.

### Behavioural Data Analysis and Task Validation

Reaction time data were lost due to equipment failure. Accuracy data were submitted to a repeated measures analysis of variance (ANOVA) with four factors (visual field, response hand, stimulus type, and stimulus format). The significance threshold was set at p < 0.05. Post-hoc paired *t*-tests were performed where appropriate (Bonferroni correction).

Accuracy data are presented as mean +/- standard error. Overall, participants were able to perform the task accurately (82.0 +/- 1.6%). There was a main effect of visual field (F(1,23) = 7.50, p < 0.05), with greater accuracy in the RVF (83.2 +/- 1.8%) than the LVF (80.8 +/- 1.6%). This effect can be explained by the visual field by type interaction (F(1,23) = 6.78, p < 0.05). Post-hoc tests revealed greater accuracy for word stimuli in the RVF than the LVF (t(23) = 3.30, p < 0.05, Bonferroni corrected), with no significant effects of visual field for face stimuli. Finally, there was a main effect of format (F(1,23) = 17.49, p < 0.001), with significantly more accurate responses for words and faces (86.9 +/- 1.6%) than pseudowords and scrambled faces (77.1 +/- 2.3%). In summary, there was an effect of visual IT for word stimuli, highlighting the interaction between visual IT and stimulus type.

### Imaging

Imaging was performed on a 4 T magnet (Oxford Magnet Technology) using an INOVA™ console (Varian, Inc.), 36 mT/m imaging gradients (Tesla Engineering), and a transverse electromagnetic quadrature radiofrequency coil (Bioengineering, Inc.). A two-shot spiral sequence was used to acquire whole brain fMRI data (repetition time [TR] = 1000 ms, effective TR = 2000 ms, echo time [TE] = 30 ms, flip angle = 60 degrees). Twenty axial slices (6 mm thick, 0.6 mm gap) were acquired with a 240 × 240 mm field of view (FOV) and a 64 × 64 matrix. For each sub-run, 165 volumes were acquired, for a total of 660 volumes per participant. At the beginning of each sub-run, four dummy scans preceded the acquisition. A navigator echo and a field map correction were applied during image reconstruction. A whole brain, T1-weighted anatomical image was also acquired using a magnetization prepared fast low angle shot (MPFLASH) sequence (TR = 10 ms, TE = 5 ms, inversion time = 500 ms, flip angle = 11 degrees, 240 × 240 mm FOV, 256 × 128 matrix, 203 axial slices, 0.94 mm thick).

### Imaging Data Analysis

#### Spatial Pre-processing

Statistical Parametric Mapping '05 (SPM5) (Wellcome Department of Cognitive Neurology, Institute of Neurology) [56,57] running on Matlab 6.5.1 (R13) was used for pre-processing and statistical analysis. Images were realigned to correct for head motion using a six parameter, rigid body transformation. A two pass procedure was employed: images were initially realigned to the first image and then registered to the mean image. The participant's anatomical image was then coregistered to their functional images, using the mean functional image as the reference. To transform the data to Montreal Neurological Institute (MNI) space, the mean functional image was warped to a functional image template using an optimized 12 parameter affine transformation followed by a series of nonlinear deformations. This transformation was then applied to all volumes (voxels resampled to 3 × 3 × 3 mm) and the coregistered anatomical image. The functional data were spatially smoothed using a 7.5 mm isotropic field-width-at-half-maximum Gaussian kernel.

#### Statistical Analyses

A global scaling factor was used to account for signal drift between scanning sessions within each subject. A highpass filter (period: 128 s) was applied. An autoregressive (order 1) plus white noise model was used to account for short range serial temporal correlations [45,58]. Regressors for modelling the observed responses were created by convolving stimulus onset vectors for each condition with SPM5's hemodynamic response function (HRF) and its time and dispersion derivatives [59]. Six movement parameters (output from the realignment procedure) were included as regressors of no interest. For each subject, *t*-contrasts of the HRF regressors were created to compare activation associated with each condition against baseline. *T*-contrasts were also created to compare conditions (e.g., high IT > low IT). Unless otherwise stated, the activation threshold was p < 0.05 (FWE corrected, extent = 2). To assign anatomical labels to activations, the MNI coordinates were converted to Talairach space using a nonlinear transformation [60] and anatomically identified using the Talairach Daemon Client 2.0 [61,62] and the MNI Space Utility [63].

At the group level, a random-effects analysis was employed. For each *t*-contrast, an ANOVA was performed with three factors: the HRF regressor and its time and dispersion derivatives. Group effects were then assessed with *t*-contrasts for the HRF regressor (time and dispersion derivatives were factors of no interest).

For the individual level analysis, whole brain task related activation was examined. To identify a cluster as callosal activation, the majority of the cluster's voxels had to be located in the corpus callosum (defined by a region of interest [ROI] created in WFU PickAtlas [64]). This reduced the potential contribution of partial volume effects (i.e., clusters that were located primarily in gray matter were excluded).

In addition, voxels with significant movement related activity were also excluded. Movement related signal changes were identified by performing an *F*-test on the motion regressors (p < 0.05, FWE corrected, extent = 2). Two of the five subjects with callosal activation had overlapping movement related activity (S03: 14 out of 66 voxels; S04: 1 out of 23 voxels). These voxels were excluded from the description of the callosal activation (Table 1) and the time course analysis (Figures 3 and 4; see below). In addition, a sixth subject had task-related callosal activation, but was excluded because all of the activated callosal voxels had significant movement-related activity.

#### Time Course Analysis

Time course data were extracted from ROIs based on both functional and structural boundaries using MarsBaR [65]. The white matter ROIs were derived from the corpus callosum clusters (described above). The gray matter ROIs were derived from activation clusters that were the best match to the corpus callosum activation clusters in terms of intensity and extent (Table 1). White matter voxels were excluded from these clusters by performing an intersection with the subject's gray matter segmentation map. For one subject (S03), the most comparable gray matter cluster was much larger than the callosal cluster. In this case, a subset of gray matter voxels was selected by performing another intersection with a partially overlapping cortical region (defined using the WFU PickAtlas such that the resulting ROI was approximately matched for extent with the white matter ROI). Signals were extracted from all four sub-runs and demeaned to account for global signal differences. The sub-runs were averaged together for each subject. The frequency spectra of the time courses were created using fast Fourier transforms (FFT).

## Authors' contributions

ELM and RCND contributed the experimental design. ELM, RCND, and SDB performed the data collection and interpretation of the results. ELM performed the data analysis with input from RCND and SDB. ELM and RCND drafted the manuscript. All authors read and approved the final manuscript.

## Supplementary Material

Additional file 1 **Whole brain activation (group level)**. Activation maxima for the four conditions (p < 0.05, FWE corrected, extent = 2). L: left, R = right. Click here for file
